# The complete mitochondrial genome of *Alphitobius diaperinus* Panzer, 1797 (Coleoptera: Tenebrionidae)

**DOI:** 10.1080/23802359.2020.1772684

**Published:** 2020-06-02

**Authors:** Ki-Jeong Hong, Woong Ki, Doo-Sang Park, Byung-Kun Yang, Hyobin Lee, Jongsun Park, Wonhoon Lee

**Affiliations:** a Department of Plant Medicine, Sunchon National University, Suncheon, Korea;; b Biological Resource Center, KRIBB, Jeongeub, Korea;; c Biogenoci Co., Ltd, Suwon, Korea;; d Department of Plant Medicine, Gyeongsang National University, Jinju, The Republic of Korea;; e Infoboss Co., Ltd, Seoul, Republic of Korea;; f InfoBoss Research Center, Seoul, Republic of Korea;; g Institute of Agriculture and Life Science, Gyeongsang National University, Jinju, The Republic of Korea

**Keywords:** Mitochondrial genome, *Alphitobius diaperinus*, Coleoptera, Tenebrionidae, South Korea

## Abstract

*Alphitobius diaperinus* Panzer, 1797 is a major pest in poultry production and easily observed in poultry litter. We have determined mitochondrial genome of *A. diaperinus* collected in Chungcheongbuk-do, Republic of Korea. The circular mitogenome of *A. diaperinus* is 15,511 bp long which is longer than that of *Z. atratus* but shorter than that of *T. obscurus*. It includes 13 protein-coding genes, two ribosomal RNA genes, and 22 transfer RNAs. The base composition was AT-biased (72.4%). Phylogenetic tree displays that tribe Alphitobiini is clustered with tribes Helopini and Diaperini with enough supportive values of three phylogenetic trees.

The darkling beetle, *Alphitobius diaperinus* Panzer, 1797 (Coleoptera: Tenebrionidae), is a significant pest in poultry worldwide. Though these beetles are small, their importance as a poultry pest is enormous. It is also considered a primary structural pest in the poultry industry, causing extensive damage to broiler housing, which has led to increased heating and repair costs for poultry producers (Axtell and Arends 1990).

To understand its genetic background, we completed mitogenome of *A. diaperinus*, as the first mitochondrial genome in tribe Alphitobiini, collected in Daeso-myeon, Eumseong-gun, Chungcheongbuk-do, Republic of Korea (36°96′77″N, 127°51′61″E; the specimen and its DNA were deposited at the Sunchon National University, Korea; Accession number: 190925HK004). DNA was extracted using DNeasy Blood &Tissue Kit (QIAGEN, Hilden, Germany). Raw sequences from Illumina HiSeqX (Macrogen, Korea) were filtered by Trimmomatic 0.33 (Bolger et al. 2014) and *de novo* assembled using Velvet 1.2.10 (Zerbino and Birney 2008), SOAPGapCloser 1.12 (Zhao et al. 2011), BWA 0.7.17 (Li 2013), and SAMtools 1.9 (Li et al. 2009). Geneious R11 11.1.5 (Biomatters Ltd, Auckland, New Zealand) was used to annotate based on several mitogenomes including *Zophobas atratus* (NC_041101; Bai et al. 2019), *Tenebrio obscurus* (NC_037196; Bai et al. 2018), and *Tenebrio molitor* (NC_024633; Li-Na and Cheng-Ye 2014).

*Alphitobius diaperinus* mitogenome (GenBank accession is MT165524) is 15,511 bp long, which is longer than that of *Z. atratus* (15,494 bp) but shorter than those of *T. obscurus* (15,771 bp) and *T. molitor* (15,785 bp). It contains 13 protein-coding genes (PCGs), 37 tRNAs, and two rRNAs. The base composition was AT-biased (72.4%) and gene order was identical to 23 available Tenebrionidae mitogenomes.

We inferred the phylogenetic relationship of 24 Tenebrionidae mitogenomes, including *A. diaperinus* mitogenome. Complete mitochondrial genomes were aligned by MAFFT 7.450 (Katoh and Standley 2013) after rearranging sequences including utilizing reverse complement sequences and moving unaligned sequences to the end of mitogenomes. Multiple sequence alignment was used for constructing bootstrapped neighbor-joining, maximum-likelihood, and Bayesian inference phylogenetic trees with MEGA X (Kumar et al. 2018) and Mr. Bayes (Huelsenbeck and Ronquist 2001), respectively. Phylogenetic tree displays that tribe Alphitobiini is clustered with tribes Helopini and Diaperini with enough supportive values of three phylogenetic trees (Figure 1). Some of higher clades not supported by three phylogenetic trees (see bold supportive values in Figure 1) indicate that the phylogenetic relationship of species in Tenebrioninae should be investigated more with additional mitochondrial genomes which will be available in near future. In addition, three mitochondrial genomes of *Tribolium castaneum* display high supportive values of three phylogenetic trees, which is similar to those of *Laodelphax striatellus* (Park, Jung, et al. 2019; Seo et al. 2019) and *Stegobium paniceum* (Park et al., under review); while it is different from those of *Nilaparvata lugens* (Choi et al. 2019; Park, Kwon, et al. 2019; Choi et al., under review).

**Figure 1.**
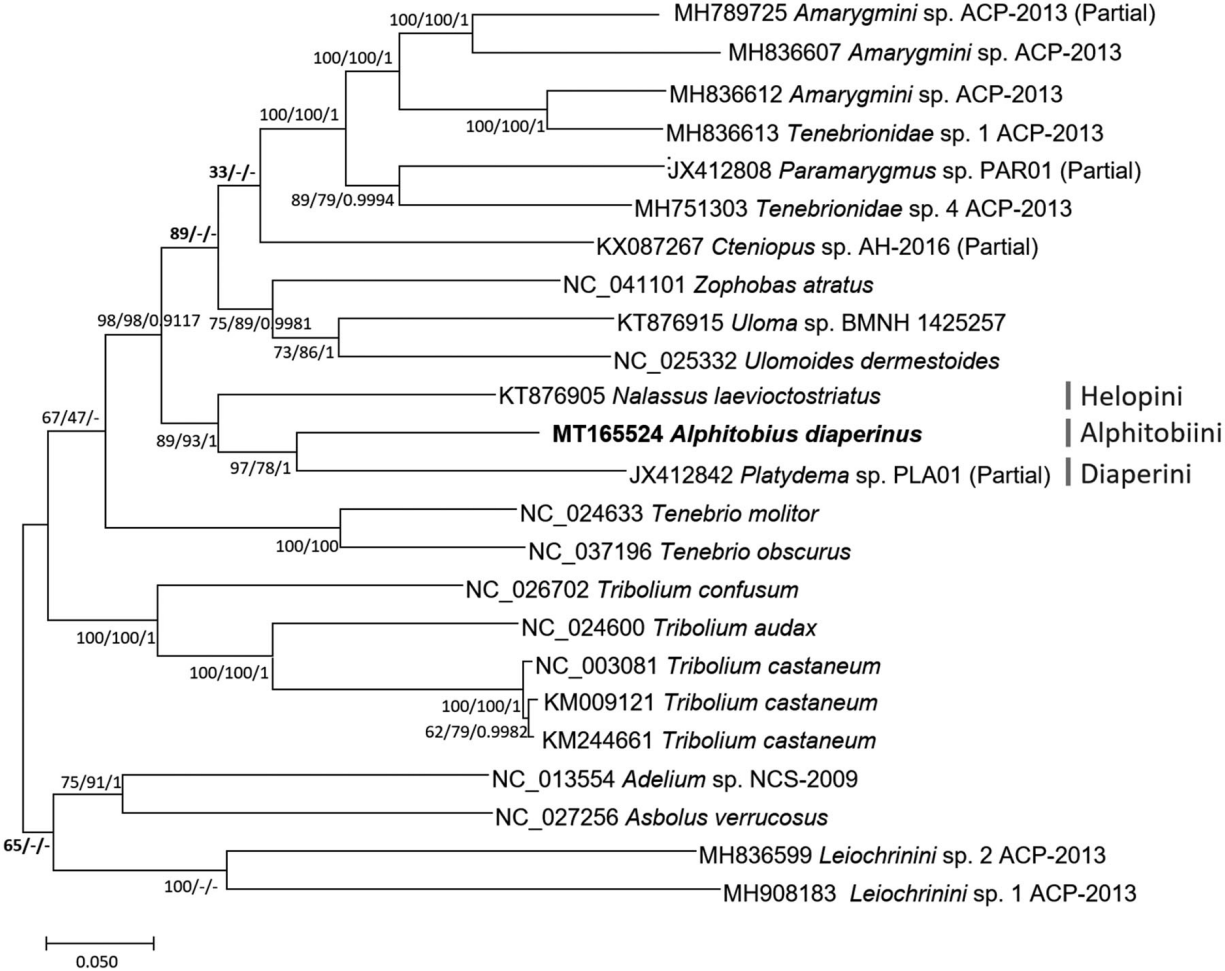
Bayesian inference (1,000,000 generations), maximum-likelihood (1000 bootstrap repeats), and neighbor-joining (10,000 bootstrap repeats) phylogenetic trees of 24 Tenebrionidae mitochondrial genomes: *Alphitobius diaperinus* (MT165524), *Amarygmini* sp. (MH789725; Partial mitochondrial genome; MH836607, and MH836612), *Tenebrionidae* sp. (MH836613 and MH751303), *Paramarygmus* sp. (JX412808; Partial mitochondrial genome), *Cteniopus* sp. (KX087267; Partial mitochondrial genome), *Zophobasatratus* (NC_041101), *Uloma* sp. (KT876915), *Ulomoides dermestoides* (NC_025332), *Nalassus laevioctostriatus* (KT876905), *Platydema* sp. (JX412842), *Tenebrio molitor* (NC_024633), *Tenebrio obscurus* (NC_037196), *Tribolium confusum* (NC_026702), *Tribolium audax* (NC_024600), *Tribolium castaneum* (NC_003081, KM009121, and KM244661), *Adelium* sp. (NC_013554), *Asbolus verrucosus* (NC_027256), and *Leiochrinini* sp. (MH836599 and MH908183). Phylogenetic tree was drawn based on maximum-likelihood tree. The numbers above branches indicate bootstrap support values of maximum-likelihood and neighbor-joining phylogenetic trees and posterior probability value of Bayesian inference tree, respectively. Tribe names were displayed as light gray color. Bolded supportive values indicate nodes that all three phylogenetic trees show different topologies.

## Data Availability

The data that support the findings of this study are openly available in NCBI (National Center for Biotechnology Information) at https://www.ncbi.nlm.nih.gov/nuccore/MT165524.
